# Cardiovascular screening in general practice in a low SES area

**DOI:** 10.1186/1471-2296-13-117

**Published:** 2012-12-10

**Authors:** Ans H Tiessen, Andries J Smit, Sebes Zevenhuizen, Edwin M Spithoven, Klaas Van der Meer

**Affiliations:** 1 University of Groningen, University Medical Center Groningen, Dept. General Practice, Groningen, The Netherlands; 2 University of Groningen, University Medical Center Groningen, Dept. Internal medicine, Groningen, The Netherlands; 3 General practitioner, Oude Pekela, Groningen, The Netherlands

**Keywords:** General practice, Socio-economic status (SES), Cardiovascular risk management, Screening

## Abstract

**Background:**

Lower social economic status (SES) is related to an elevated cardiovascular (CV) risk. A pro-active primary prevention CV screening approach in general practice (GP) might be effective in a region with a low mean SES. This approach, supported by a regional GP laboratory, was investigated on feasibility, attendance rate and proportion of persons identified with an elevated risk.

**Methods:**

In a region with a low mean SES, men and women aged ≥50/55 years, respectively, were invited for cardiovascular risk profiling, based on SCORE 10-year risk of fatal cardiovascular disease and additional risk factors (family history, weight and end organ damage). Screening was performed by laboratory personnel, at the GP practice. Treatment advice was based on Dutch GP guidelines for cardiovascular risk management. Response rates were compared to those in five other practices, using the same screening method.

**Results:**

521 persons received invitations, 354 (68%) were interested, 33 did not attend and 43 were not further analysed because of already known diabetes/cardiovascular disease. Eventually 278 risk profiles were analysed, of which 60% had a low cardiovascular risk (SCORE-risk <5%). From the 40% participants with a SCORE-risk ≥5%, 60% did not receive medication yet for hypertension/hypercholesterolemia. In the other five GPs response rates were comparable to the currently described GP.

**Conclusion:**

Screening in GP in a low SES area, performed by a laboratory service, was feasible, resulted in high attendance, and identification and treatment advice of many new persons at risk for cardiovascular disease.

## Background

Management of cardiovascular risk factors in high-risk persons in general practice is still poor, as shown by the EUROASPIRE III 2006–2007 survey in 12 European countries [1]. In the United Kingdom (UK) Joint British Societies JBS 2 guidelines were proposed in 2005 for prevention of cardiovascular disease (CVD) in the asymptomatic population in clinical practice [2]. Recording of CVD risk factors was also stimulated by the introduction of the Quality and Outcomes Framework (QOF) in 2004. The implementation of the National Health Service Health Checks by the UK Department of Health has been started in 2009, but has major implications for the workload of general practices [3], especially due to variable, but often incomplete baseline risk factor recording. In several other countries initiatives have been taken to assess the potential of general practice to improve vascular disease prevention, such as the HIPS study in Australia [4] and a CVD quality improvement programme in Finland [5]. Population based screening for cardiovascular disease has also been studied in the UK using routinely available data from GP records (Epic-Norfolk study) and was shown to perform reasonably well [6]. Other approaches involve different health care suppliers such as community pharmacies [7]. Still, the large majority of primary prevention occurs in the primary care setting in general practices.

Up until recently, in Dutch general practice guidelines a case finding approach was used for primary CVD prevention. Recently however, a so-called ‘Prevention Visit’ approach for use in general practice has been introduced and tested in the Netherlands [8,9]. 82% of the Dutch population between 45–74 years prefers GP-based health checks for cardio metabolic risk [10]. For this Prevention Visit, persons >45 years were invited to fill in a self-report questionnaire to identify high risk individuals. Response rates depended on the way persons were approached, but were generally found to be poor to moderate [9]. From the individuals invited by a personal letter, followed by a reminder-letter, 33% filled in the questionnaire and from the target population invited by poster or leaflet in the GP waiting room, 1% filled in the questionnaire. After this, medical consultation by general practitioners was offered to the identified high risk individuals. This consultation consisted of comprehensive CVD risk factor assessment and treatment advice. Acceptance by general practitioners of cardio metabolic health checks is still slow, and may like the NHS Health Checks suffer from the considerable time and work load they impose.

Several studies, for example from Sweden, USA, France and the UK, have investigated the relation between socio-economic status (SES) and cardiovascular disease. Despite large regional differences between these studies, with the SES based on educational level and/or occupational group and CVD expressed as mortality, morbidity and/or presence of risk factors, these studies all found that higher SES is associated with lower CVD risk [11-16]. Individuals with low SES more strongly prefer CVD screening by their own GP (63% compared to 49% of the individuals with high SES, p<.001) [10]. Perhaps, an active personal approach coming from their own GP may be most successful for this target group.

In the present article, results are reported for CVD risk factor detection and advice, antedating but partly similar to the Dutch Prevention Visit described above. Major differences with the Prevention Visit were that possible participants were actively approached by invitations on behalf of their GP (without prior selection based on a questionnaire), and a regional GP laboratory service and personnel performed the risk factor assessment within the GP setting, to reduce workload for the GP. A third difference is that for each participant, an individual GP guideline-based treatment and follow-up advice was given by the GP laboratory service, but its execution was left to the discretion of the GP. This collaboration between laboratory services and GP practices existed already in several Dutch regions for the treatment of diabetes and showed improvements on control frequency and glycaemic control with this intervention compared to controls [17,18]. In this kind of collaboration, GP laboratories usually offer a recall system for regular patient check-ups, the results of which are reported to the GP.

This study was performed in a general practice in a municipality with a low SES status (1.6 standard deviation below the Dutch average, based on income, employment and level of education of the postal code region) [19] and markedly increased cardiovascular mortality (the standardised mortality ratio is 124 (95% CI: 106–143), which means that CVD mortality standardised for age is 24% higher than the Dutch average level) [20].

In the current report we addressed the following questions on the pro-active GP based approach, supported by a GP laboratory, in a low SES-area: what are the response rate and attendance rate at the screening of the invitees and what are the reasons for non-attendance? How many of those with risk profiles that were selected for further analysis, appeared to be “new high risk patients” i.e. high risk CVD patients without previously receiving CVD-medication? Comparisons of response and attendance rates are made with summary data for the same approach in other municipalities. From these other municipalities, one was located in a substantial higher SES municipality. The yield of high risk patients for the studied low mean SES municipality has been compared to the yield in this higher SES municipality. The comparison with this other practices was performed to assess the external validity of the screening. Our hypothesis is that with this approach the response rate and attendance rate of the low SES municipality will equal the other municipalities and we expect that the yield of high risk patients will be higher in the low SES municipality compared to a higher SES municipality.

## Methods

### Selection of participants

The screening was performed between April and September 2008 in a general practice in Oude Pekela, Eastern Groningen, with a population size of 2850 patients (the average GP practice number in the Netherlands is 2350).

Male persons from 50 years on and females from 55 years on, without registered diabetes mellitus, were preselected from this population using the computer system for patient registration. Asylum seekers (80 persons in this practice) were not approached because follow-up often was not possible. The general practitioner could furthermore exclude persons for whom participation was considered inappropriate, for example patients suffering severe dementia or persons with an impaired life expectancy (estimated <2 years), together 13 persons in this practice, or incapacity to come to the general practice due to a very old age or an enduring bedridden condition, 24 persons. Current follow-up by a cardiologist or internist (second line) was also an exclusion criterion. Persons already receiving CV risk factor treatment (except diabetes) in a primary prevention setting were included. Those with previous cardiovascular events, but not in second-line follow-up, were invited for possible updating/ optimisation of secondary prevention. This latter group was not taken into account in the further analysis below.

The selected persons received an information letter and a response form on behalf of their general practitioner. Respondents willing to participate all received an invitation and a questionnaire (Additional file 1).

Each invitee received an appointment at the location of the general practice, scheduled for half an hour. The filled-in questionnaire was discussed with the representative of the general practice service LabNoord Groningen, usually a doctor’s assistant. This representative had previously received additional training for taking the cardiovascular history and physical examination. Specific attention was given to performing blood pressure measurements according to the guidelines.

The previously sent questionnaire consisted of the following items: previous history and family history of cardiovascular disease, hypertension, diabetes, lipid disorders, medication use, smoking behaviour and physical exercise habits. The physical examination included length, weight, BMI, and standardised blood pressure measurement. Blood pressure was measured three times on both arms; the mean of three measurements on the arm with the highest blood pressure was used. Additionally a 12-lead ECG, an albumin-creatinine ratio in a morning urine sample, and non-fasting serum assays of creatinine (with the estimated glomerular filtration rate (eGFR)), glucose, potassium, total cholesterol, HDL-cholesterol, LDL-cholesterol and triglycerides were assessed. The ECG was examined by a cardiologist.

Using the collected data, for each attendant to the screening, a report was composed for the general practitioner by the GP laboratory service LabNoord. This summarised the identified risk factors, the calculated SCORE risk assessment (ten-year cardiovascular mortality risk) [21], and included advices for lifestyle and pharmaceutical treatment, for possible second-line specialist referral advices, and for follow-up by the general practitioner himself or LabNoord. The advices were based on the SCORE assessment plus additional risk factors, in line with the Dutch GP guideline [22]. Additional risk factors were: positive family history of first degree relatives with cardiovascular disease <60 years, overweight (BMI >27 kg/m^2^), symptoms of end organ damage such as micro albuminuria (albumin/creatinine ratio: ♀>3,5 /♂>2,5), eGFR <60 ml/min/1.73 m^2^, or left ventricular hypertrophy on ECG. SES is not part of the SCORE risk calculation. A vascular internist examined the data and gave lifestyle pharmaceutical and follow-up advices based on the Dutch cardiovascular risk management GP guideline. The general practitioner received this report approximately 2 weeks after the screening visit, earlier in case of abnormal results requiring more urgent action. Depending on the results and the advice, the screened person received a letter or call for a GP visit to implement the lifestyle or pharmaceutical advices and follow-up as chosen by the GP.

The screening performed by the general practitioner in cooperation with the general practitioner’s laboratory was not registered at a medical ethics committee. Comparable cooperative screening facilities between general practitioners and the laboratory already existed several years for diabetes mellitus and COPD. The participating general practitioner in this study and the laboratory wanted to extend these screening facilities, in the interest of the local patients. The region of the general practice is known for its elevated cardiovascular risk [20] and in general patients increasingly demand medical screenings. With the current methods, experiences from the diabetes screening could be used to offer a screening programme embedded in a well-organised setting with quality insurance including an appropriate follow-up. Afterwards it was decided to publish the findings of this - at least for the Netherlands - novel initiative. All the procedures on the subjects as described in our article were part of usual risk factor detection, according to the Dutch cardiovascular risk management guidelines.

141 persons who did not respond or refused participation were asked half a year later by letter for the reasons not to respond or participate.

As mentioned in the backgrounds, this study was performed in a general practice in a municipality with a SES status 1.6 standard deviation below the Dutch average, based on income, employment and level of education of the postal code region [19]. We analysed whether the postal code of the attendees was different from the whole practice (excluding the asylum seekers), using Fisher’s exact test 2-sided, to assess if these further analysed participants reflected the low SES population of the practice.

After the first screening, the same approach was used in five other general practices that were also already collaborating with LabNoord for the treatment of diabetes. Four from these other practices were located at the town of Stadskanaal, located in a municipality with a mean SES of 1.4 standard deviation below the Dutch average [19]. The fifth practice was located at Drachten, municipality Smallingerland, with a mean SES of 0.6 standard deviation below the Dutch average, which is substantially higher than the other practices [19]. Summary response rates of all five practices were compared to the described practice, using Fisher’s exact test 2-sided. The yield in identified persons with an elevated risk in the studied practice has been compared to the general practice from the higher SES municipality. For this municipality the cardiovascular mortality is at a level comparable to the average in the Netherlands (with a standardised mortality ratio of 105 (95% CI: 97 – 113), which is substantially higher than the studied practice [23].

## Results

**Figure 1:**
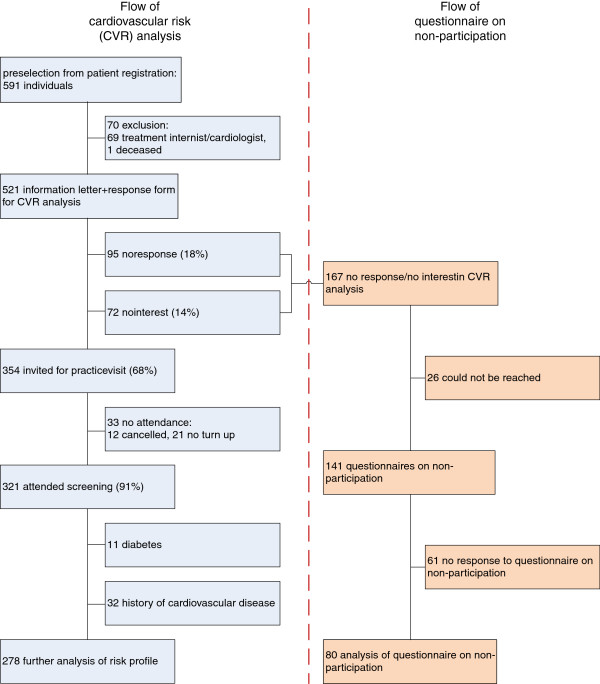
Flow of patients.

As shown in Figure 1, 591 individuals were preselected from the patient database. These were individuals in the requested age category, being no asylum seeker and without registered diabetes or conditions considered inappropriate for participation. In the next step 69 individuals being under second line follow up and 1 person that had deceased were excluded.

521 persons were sent a letter inviting them for participation. 18% did not respond, 14% were not interested in participation and 68% responded positively and were invited for a screening visit. 91% of these invitees (which is 62% of all persons that were sent an invitation letter) attended the screening. 11 persons invited for the screening appeared to have previously had an increased glucose level but had not been noted in the practice registry as having diabetes. 32 persons had suffered previous cardiovascular events. The risk profiles of these latter two groups were assessed but not used in the further analysis below. The remaining 278 risk profiles were analysed further.

**Figure 2:**
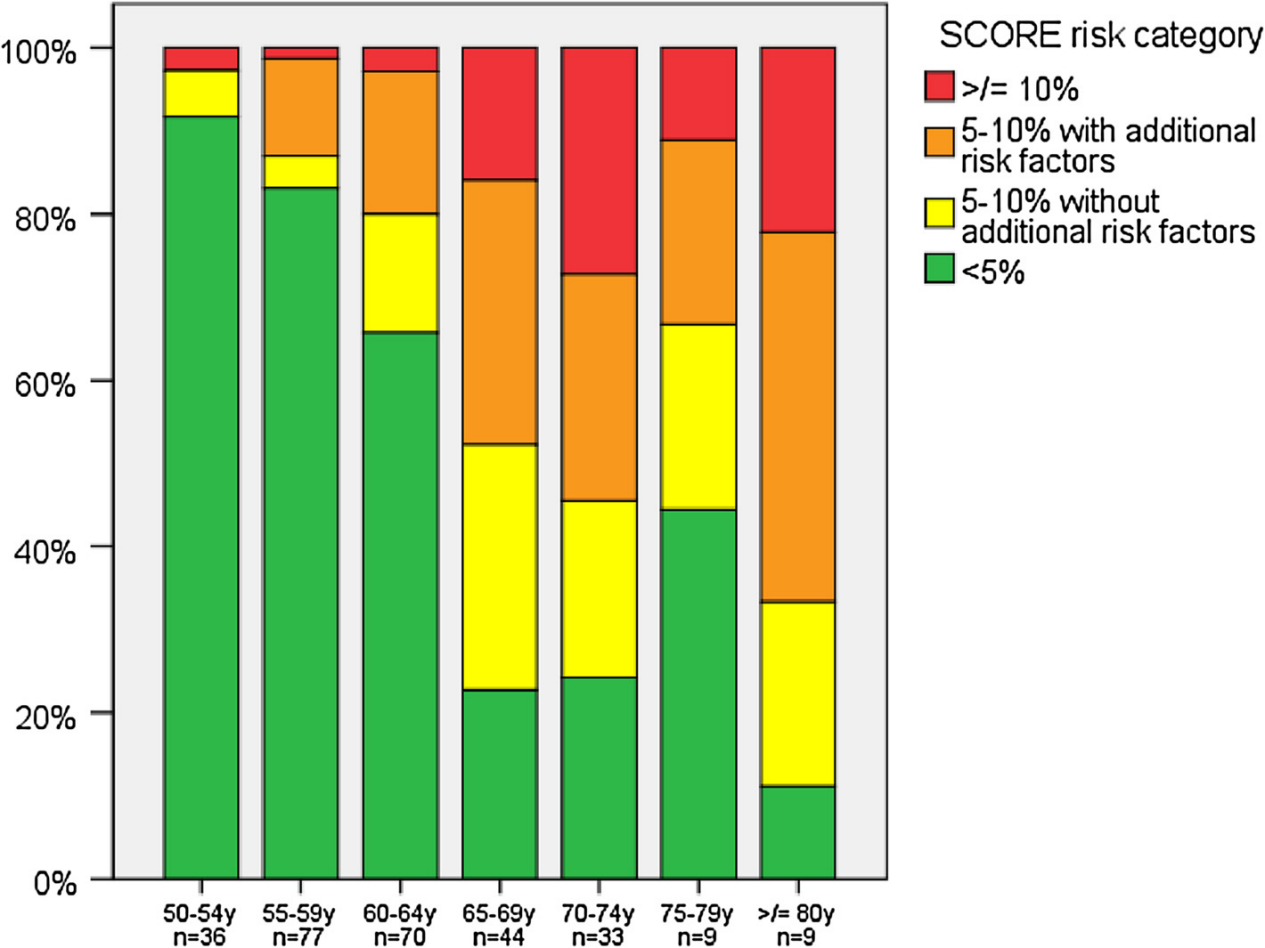
Distribution of SCORE risk categories for different age groups in the studied general practice.

From these 278 analysed risk profiles, 59.7% (95% CI: 53.7 – 65.5, n=166) had a low cardiovascular risk, being in the category <5% ten-year cardiovascular mortality risk. 30.1% (95% CI: 23.3 – 37.7, n=50) of this low risk group used pharmaceutical treatment for hypertension and/or hypercholesterolemia, and were known using this treatment in the general practice. Below the age of 65 years, 78.1% (143/183; 95% CI: 71.4 – 83.9) had a low risk (<5%), between 50 and 55 years (only men) this was 91.7% (33/36; 95% CI: 77.5 – 98.2) (Figure 2).

14.0% (95% CI: 10.2 – 18.7, n=39) of the 278 analysed participants had an intermediate cardiovascular risk (5-10% without additional risk factors), and 26.3% (95% CI: 21.2 – 31.8, n= 73) had an increased risk (5-10% with additional risk factors or ≥10%). Of the complete group with a risk ≥5%, 40.2% (45/112; 95% CI: 31.0 – 49.9) was already being treated for hypertension or hypercholesterolemia, but was nevertheless in the intermediate or high risk group. 24.1% (95% CI: 19.2 – 29.6, n = 67) of all 278 analysed participants were individuals with a (moderately) increased risk (≥5%), without previously receiving pharmaceutical treatment for hypertension or hypercholesterolemia.

95% of all analysed risk profiles and 93% of the whole general practice population (p=.22) lived in the postal code region with the SES status −1.6 SD below the mean of the Netherlands [19].

80 of 141 questionnaires on non-participation were returned, see Figure 1. Given reasons for non-participation were combinations of not being convinced of usefulness, the wish not to know the CV risk, being afraid of unwanted results (24), lack of time (16), and financial costs (13). The remaining category (27) mentioned reasons including: age, other health problems, missed call or combinations of these.

Response rates and yield for use of the current model in five other general practices in other cities and regions in the northern part of the Netherlands, also in a higher SES class region are shown in Table 1 (Ref: unpublished data from the CV screening at Drachten and Stadskanaal, LabNoord Groningen; 2009). Compared with the 1874 initially addressed persons in these other practices, the proportion of person who were not interested was slightly lower (13.8% vs. 18.8%) and related to this, the proportion invitees was slightly higher (67.9% vs. 62.1%) in the studied practice, with the proportions being in the range that was also found for the other practices. The yield in identified persons with an elevated risk has been compared to one of these practices, which is located in a city with a cardiovascular mortality at a level comparable to the average in the Netherlands [23], which is considerably lower than in the currently reported low SES municipality. In this practice in the area with a lower cardiovascular risk, 29.6% (95% CI: 23.4 – 36.5) was found to have a SCORE risk assessment ≥5% compared to 40.3% (95% CI: 34.5 – 46.3) in the current study group; with the difference between both proportions being 10.6% (95% CI: 2.06 – 19.20).

**Table 1:** Proportions of non-responders, individuals who were not interested, invitees and attenders at the screening for the described GP (“Oude Pekela”), compared to the 5 other GPs

	**Oude Pekela GP (521 information letters) N (%)**	**Similar screening in 5 other GPs (total 1874 information letters)**		**Difference between Oude Pekela and 5 other GPs P-value**
		**N (%)**	**Highest-Lowest GP**	
**No response**	95 (18.2)	358 (19.1)	10% – 32%	.70
**No interest**	72 (13.8)	353 (18.8)	13% – 27%	.01
**Invitation**	354 (67.9)	1163 (62.1)	51 – 72%	.01
**Attendance at screening (% of invitees)**	321 (90.7)	1090 (93.7)	88% – 100%	.06

## Discussion

This primary prevention CV risk screening, performed within the general practice with logistical support of the regional general practice laboratory and service, could be well managed within the GP setting, and resulted in a limited additional workload for the GP and his assistant. The GP was only involved in the selection of the patients and provided the location within his practice. The response rate and attendance of the invitees was high, when compared to the Dutch Prevention Visit for example. The yield in newly identified persons (i.e. not currently treated already for hypertension or hypercholesterolemia) with increased risk was considerable (24% of the analysed participants). Compared to a practice from an area with a lower cardiovascular mortality, the yield in identified high risk patient was higher in the studied practice, as expected. Compared to the five practices with similar screenings, the proportion of individuals that was interested after written invitation and could be invited for the risk assessment, was even slightly higher in the studied practice.

The percentage of individuals that had received an invitation letter and was eventually invited for the screening (68%) is comparable with the results of the Dutch ResCon research agency, which assessed by questionnaires in 45–74 year old persons their willingness to participate in a health check for early detection of cardio metabolic risk: 66% reported to probably or surely be willing to undergo a health check [10]. Likely reasons for the good attendance are the invitation by their own general practitioner, the screening location within the general practice and the reimbursement by the health insurance provider (depending on the remaining own risk) [10]. The response rate was considerably higher than during the pilot of the Dutch national Prevention Visit. The admission age was higher in the present study, which is related to a higher response [24-26]*.* For example, Van de Kerkhof et al. found that respondents at a cardiovascular risk factor screening were significantly older than non-responders (52.7 vs. 49.5 years, p<.001) [24]. Besides, a considerable number of the analysed participants already used medication and thus was already a regular visitor of the practice. These people probably experience fewer barriers to participate in a medical programme.

Both in the pilot study of the Prevention Visit as well as in the study by Van de Kerkhof et al. a questionnaire was used as first selection step [9,24]. The dependence on a questionnaire to be filled in by possible candidates as a first filter for a cardio metabolic screening seems to be less efficient in identifying high risk people than increasing the admission age from 40 to 50 years [27]. In the present study even in the 50–55 years range, the large majority (92%) of men was in the low risk range. Active invitation is needed to additionally obtain information on missing or incompletely available risk factors for an adequate assessment and identification of high CVD risk patients, as is shown in our and the Prevention Visit pilot studies [9], but also in the UK primary care setting [28].

In the present municipality with a low average SES and increased mortality for cardiovascular disease, the target group had a high response rate with the current approach. Thus, an active screening as performed in the current study seems to be an answer to the demand for a reliable risk assessment in such a low SES group. The expectation that many undertreated persons with increased risk would be identified using the approach via their general practice was confirmed.

A limitation of the currently reported data of only one specific general practice situation may be that the external validity is limited. However, use of the currently described model of CV risk screening within a general practice, in several other general practices in other cities and regions in the northern part of the Netherlands, also in a higher SES class region, revealed no major differences in response rates and yield.

The participating general practitioners, both from the studied practice and from the other municipalities, may be more motivated for improving cardiovascular screening and adjusting practice organisation than average practices, as this was a novel initiative. This may overestimate the effect.

Within the 40% of the further analysed participants with a SCORE risk assessment ≥5% already using pharmaceutical treatment for the risk factors, many were not on the target levels as advised in Dutch and other national CV guidelines. Similar observations have often been made in primary as well as in secondary care [1,29]. This group received additional advice as suggested in the guidelines. It illustrates that extension of the current health check model to those in a general practice already receiving pharmaceutical treatment for cardiovascular risk factors may still have added value.

In the described current CV screening model, males were invited >50 years and females >55 years, because SCORE risk assessments are to a large extent determined by age and start to rise to levels qualifying for lifestyle or even pharmaceutical treatment above these age levels [21]. In other health check risk screening programs, such as the NHS Health Checks programme, but also in the Dutch Prevention Consultation, younger age limits such as 40 or 45 years are used [3,9]. Current JBS charts allow assessments for three age ranges: <50, 50–59 and ≥60 years. We are aware that use of the lower age limit better takes into account that younger patients have greater lifetime risk. One may also argue that lowering the admission age has the second advantage of offering a time window for benefits from lifestyle modification with an otherwise still expectant policy. However, we weighed this against a broader acceptance (at least in the Netherlands) of the SCORE risk categories as qualifiers for treatment. Using the currently used age limits, only 8% of the males between 50–55 years did have a SCORE risk assessment ≥5%.

In the current model the logistics and execution of the screening programme and health checks were performed by a regional general practice laboratory and service. Advantages are that the health checks may be performed in or in the neighbourhood of the general practices, and invitees are examined by trained personnel but still under supervision of the general practitioner. Dalton et al. [3] have already discussed the impact of the substantial extra workload of NHS Health Checks and similar screening programmes for the GPs. Involvement of GP laboratory personnel that organises and performs the screening program within the GP practice may alleviate such extra workload. In 2008 a Dutch NIVEL questionnaire addressed to 330 general practitioners revealed that 94% consider the general practice as the preferred place for detecting high cardiovascular risk patients [30,31]. A general practice laboratory was considered the preferred institution for delegation of GP tasks (33.7% agreed/agreed very much, this was the highest percentage of all given options, which were, amongst others: hospital, pharmacy, municipal public health service) [31]. Although the general practice is well-equipped to perform follow-up for pharmaceutical treatment in the detected persons with increased CV risk, a first or parallel step of lifestyle advices may require further involvement and cooperation with dieticians or physical therapists. Practice nurses within the general practice alternatively may offer integrated advice on lifestyle and pharmaceutical treatment. For long-term follow-up of treatment results health checks after follow-up periods ranging between one and five years may be repeated using the current model.

## Conclusions

Pro-active GP based cardiovascular screening, supported by a GP laboratory, in a low SES-area could be well implemented and resulted in a high response rate of invited persons, and a high yield of newly detected persons with increased risk. Apparently, in the studied practice, it is possible with the current approach to effectively detect those with increased cardiovascular risk within the target group of persons with low mean social economic status. Further research questions that have to be answered before this approach should be broadly implemented are: whether this approach is effective in other GPs in low SES-areas, whether the approach is cost-effective and which positive effects on (long term) cardiovascular risk can be achieved in the individuals found to have an elevated cardiovascular risk.

## Abbreviations

SES: Socio-economic status; GP: General practice/practitioner; SCORE: Systematic Coronary Risk Evaluation; estimated 10-year risk of fatal cardiovascular disease; CV(D): Cardiovascular (disease); BMI: Body mass index; eGFR: Estimated glomerular filtration rate; HDL/LDL: High/low density lipoprotein; ECG: Electrocardiography; CI: Confidence interval.

## Competing interests

The authors declare that they have no competing interests.

## Authors’ contributions

AS, SZ and KM designed the screening, ES performed acquisition of data at the general practice, AT and AS drafted the manuscript. All authors read and approved the final manuscript.

## Pre-publication history

The pre-publication history for this paper can be accessed here:

http://www.biomedcentral.com/1471-2296/13/117/prepub

## Supplementary Material

Additional file 1 Questionnaire (Dutch). Click here for file
