# Supplementary material for: Cardiovascular screening in general practice in a low SES area
Source: BMC Fam Pract. 2012 Dec 10;13:117. doi: 10.1186/1471-2296-13-117 (PMC3564938; doi:10.1186/1471-2296-13-117)
Supplement: Additional file 1 — Questionnaire (Dutch). [file 1471-2296-13-117-S1.doc]

**Vragenlijst risico inventarisatie**

**Persoonsgegevens**

| Naam man/vrouw  Adres  Postcode  Woonplaats  Telefoon  Geboortedatum Leeftijd  BSN nummer  Zorgverzekeraar Inschrijfnummer |
| --- |

***Veel vragen zijn bekend bij uw huisarts. Toch vragen wij u om deze lijst zo volledig mogelijk in te vullen.***

1. **Hart en vaatziekten:**

Bent u ooit voor een van de onderstaande ziekten behandeld ? *(kruis aan wat voor u van toepassing is*)

- hartinfarct
- pijn op de borst veroorzaakt door het hart
- beroerte/TIA / herseninfarct
- pijn in de benen tijdens het lopen veroorzaakt door vernauwing in de bloedvaten van de benen
- operatie en/ of een dotterbehandeling aan de bloedvaten van het hart
- operatie en/ of dotterbehandeling van de bloedvaten in het been
- Nee
- Onbekend

1. **Familiaire ziektegeschiedenis**

Komen er hart en vaatziekten voor in uw familie **voor** het 60^ste^ levensjaar? zo ja, welke ziekte en bij wie? (ouders, broers of zusters)

- Ja, …………………………………………………………………………………………………
- Nee
- Onbekend

1. **Hoge bloeddruk**

Is er ooit bij u een te hoge bloeddruk gemeten?

- Ja
- Nee

Wordt u voor de hoge bloeddruk behandeld?

- Ja
- Nee

Komt hoge bloeddruk in uw familie voor? Zo ja bij wie ( ouders, broers, zusters)

- Ja……………………………………………………………………………………………………
- Nee
- Onbekend

Heeft u tijdens de zwangerschap een hoge bloeddruk gehad?

- Ja
- Nee

1. **Suikerziekte**

Is er ooit bij u een te hoog bloedsuikergehalte gemeten?

- Ja
- Nee

Wordt u voor suikerziekte (diabetes) behandeld?

- Alleen met dieet
- Ja, met tabletten
- Ja, met insuline
- Ja met insuline en tabletten z.o.z.

1. **Cholesterol**

Is er ooit bij u een te hoog cholesterol gemeten

- Ja
- Nee

Wordt u voor een te hoog cholesterol behandeld?

- Ja □ dieet

□ medicijnen

- Nee

1. **Medicijnen**

Welke medicijnen gebruikt u? (noteer zo volledig mogelijk: de naam, hoeveel mg, hoe vaak per dag u

de medicijnen gebruikt en sinds welk jaar)

| **Medicijn** | **Mg** | **hoe vaak per dag:** | **sinds** |
| --- | --- | --- | --- |
| ***bijv.:*** *Zocor* | *20 mg.* | *1 x 2 tabletten* | *1999* |
|  |  |  |  |
|  |  |  |  |
|  |  |  |  |
|  |  |  |  |
|  |  |  |  |
|  |  |  |  |
|  |  |  |  |
|  |  |  |  |
|  |  |  |  |
|  |  |  |  |
|  |  | |  |

1. **Roken**

Rookt u ?

- Ja
- Nee

Bent u eventueel gemotiveerd om te stoppen met roken?

- Ja
- Nee
- Weet ik niet

1. **Lichamelijke activiteit**

Doet u aan sport of andere lichamelijke activiteiten? , zo ja welke sport/ activiteiten en hoe vaak per week?

- Ja, ik …………………………………………………………………………………(activiteit/sport)
  - 1-2x per week………..(minuten)
  - 2-3x per week………..(minuten)
  - 3-4x per week………..(minuten)
  - 5-6x per week………..(minuten)
  - Dagelijks……………..(minuten)
- Nee

**Wilt u dit formulier thuis invullen en meenemen naar het spreekuur?**

| **Risicoprofiel = een overzicht van voor hart- en vaatziekten relevantie risicofactoren;** |
| --- |

**Ja Nee**

Bekend met HVZ □ □

Belaste familieanamnese □ □

Hypertensie □ □

Diabetes mellitus □ □

Hypercholesterolemie □ □

Roken □ □

Overgewicht (BMI> 25) □ □

Onvoldoende lichaamsbeweging □ □

| **Lichamelijk onderzoek** | |
| --- | --- |
| Lengte | cm |
| Gewicht | kg |
| BMI |  |
| Pols | sl/m regulai.  irregulair |
| Bloeddruk links | mm / HG |
| Bloeddruk rechts | mm / HG |
| 2^de^ bloeddruk  Links/rechts | mm/HG |
| 3^de^ bloeddruk  Links/ rechts + | mm/HG |
|  |  |
